# A Non-Vector Approach to Increase Lipid Levels in the Microalga *Planktochlorella nurekis*

**DOI:** 10.3390/molecules25020270

**Published:** 2020-01-09

**Authors:** Ewa Szpyrka, Daniel Broda, Bernadetta Oklejewicz, Magdalena Podbielska, Magdalena Slowik-Borowiec, Bartosz Jagusztyn, Grzegorz Chrzanowski, Malgorzata Kus-Liskiewicz, Magdalena Duda, Janusz Zuczek, Maciej Wnuk, Anna Lewinska

**Affiliations:** 1 Department of Biotechnology, University of Rzeszow, Pigonia 1, 35-310 Rzeszow, Poland; ewaszpyrka@interia.pl (E.S.); danielbroda@wp.pl (D.B.); b.oklejewicz@gmail.com (B.O.); magdapodbiel@gmail.com (M.P.); m.slowik_borowiec@interia.pl (M.S.-B.); bartoszjagusztyn@wp.pl (B.J.); gchrzanowski@ur.edu.pl (G.C.); mkus@ur.edu.pl (M.K.-L.); 2 Bioorganic Technologies sp. z o.o., Sedziszow Malopolski, Sielec 1A, 39-120 Sielec, Poland; mduda@bioorganictechnologies.pl (M.D.); janusz@bioorganictechnologies.pl (J.Z.)

**Keywords:** microalgae, *Planktochlorella nurekis*, DNA content, biochemical profiles

## Abstract

Microalgae are freshwater and marine unicellular photosynthetic organisms that utilize sunlight to produce biomass. Due to fast microalgal growth rate and their unique biochemical profiles and potential applications in food and renewable energy industries, the interest in microalgal research is rapidly increasing. Biochemical and genetic engineering have been considered to improve microalgal biomass production but these manipulations also limited microalgal growth. The aim of the study was the biochemical characterization of recently identified microalgal strain *Planktochlorella nurekis* with elevated cell size and DNA levels compared to wild type strain that was achieved by a safe non-vector approach, namely co-treatment with colchicine and cytochalasin B (CC). A slight increase in growth rate was observed in twelve clones of CC-treated cells. For biochemical profiling, several parameters were considered, namely the content of proteins, amino acids, lipids, fatty acids, β-glucans, chlorophylls, carotenoids, B vitamins and ash. CC-treated cells were characterized by elevated levels of lipids compared to unmodified cells. Moreover, the ratio of carotenoids to chlorophyll a and total antioxidant capacity were slightly increased in CC-treated cells. We suggest that *Planktochlorella nurekis* with modified DNA levels and improved lipid content can be considered to be used as a dietary supplement and biofuel feedstock.

## 1. Introduction

As microalgae are producers of proteins, lipids, polysaccharides, pigments, vitamins and unique secondary metabolites, microalgal biotechnology has gained attention in recent decades [1,2]. Microalgae can be used for biomass production and to obtain biotechnologically important products [1,2,3]. However, only a small number of microalgal species has been evaluated in terms of their unique biochemical properties, beneficial biological activities and potential commercial applications [1]. The most promising and widely studied microalgal species are e.g., protein-rich strains used in human nutrition *Spirulina* (*Arthrospira*) and *Chlorella vulgaris*, *Dunaliella salina* a natural source of β-carotene, *Haematococcus pluvialis* a producer of astaxanthin for aquaculture feed, *Crypthecodinium* a producer of long chain polyunsaturated fatty acid (LC-PUFA) docosahexaenoic acid (DHA) and *Nannochloropsis* a producer of eicosapentaenoic acid (EPA) [1,2,3,4,5,6]. Industrial applications of some microalgal strains may be limited due to a lack of strain robustness or low productivity under outdoor conditions [6,7]. Successful process scale-up in terms of dense biomass concentrations and high biomolecule productivities requires the real-time control and optimization that may be achieved by dynamic modeling [8]. In order to achieve full processing capabilities of microalgae as cell-factories of bio-based products, several approaches have been considered, e.g., biochemical and genetic engineering [7,9,10,11,12,13]. Microalgal growth and biomass composition may be modulated by selected environmental factors such as light, temperature and availability of nutrients and minerals [8]. In general, optimal light and temperature and nutrient replete conditions are needed to achieve enhanced growth rate [8,14]. Moreover, in nutrient replete conditions, carbon is usually built to nitrogen containing macromolecules such as proteins and amino acids [14]. When growth is limited by low availability of nitrogen, carbon is mainly built to non-nitrogen containing compounds such as neutral lipids, carbohydrates and carotenoids [14]. Indeed, lipid overproduction in numerous microalgal species is achieved by nitrogen depletion [15,16]. As nitrogen depletion-mediated increase in lipid content may be accompanied by reduced growth rate and lipid productivity [16], genetic engineering is also considered to overcome such limitations [7,12]. However, genetic manipulations involve the use of foreign genetic material and construction of genetically modified organisms (GMO) that may raise safety concerns and also require additional regulations in terms of the introduction of GMO microalgae into global food market [17,18].

In 2014, a new genus and species of microalgae have been described, namely *Planktochlorella nurekis* [19]. However, data on *Planktochlorella nurekis* growth rate, biomass composition, overall productivity and potential commercial applications are limited. Thus, in the present study, we have comprehensively characterized the biochemical profiles of the microalga *Planktochlorella nurekis*. Moreover, the main aim of the study was to use the microalga *Planktochlorella nurekis* as a model and manipulate its DNA levels and investigate how changes in DNA content may modulate its functional components. A safe non-vector approach has been applied to obtain microalgal cells with increased cell size and DNA content compared to unmodified cells. Co-treatment with two natural products was considered, namely colchicine derived from the plant *Colchicum arenarium* and cytochalasin B isolated from the plant fungal pathogen *Drechslera dematioidea* (CC co-treatment) that affected polymerization of cytoskeletal proteins and in turn inhibited karyokinesis and cytokinesis resulting in elevated cell size and DNA levels. The biochemical profiles of twelve clones of CC-treated cells were analyzed, compared to wild type (WT) cells and discussed. We have shown for the first time that CC co-treatment may result in improved lipid production, increased ratio of carotenoids to chlorophyll a and augmented total antioxidant capacity compared to unmodified microalgal cells.

## 2. Results and Discussion

### 2.1. The Effect of Co-Treatment with Colchicine and Cytochalasin B on Morphology, Cell Size and DNA Content of the Microalga Planktochlorella nurekis

**Figure 1:**
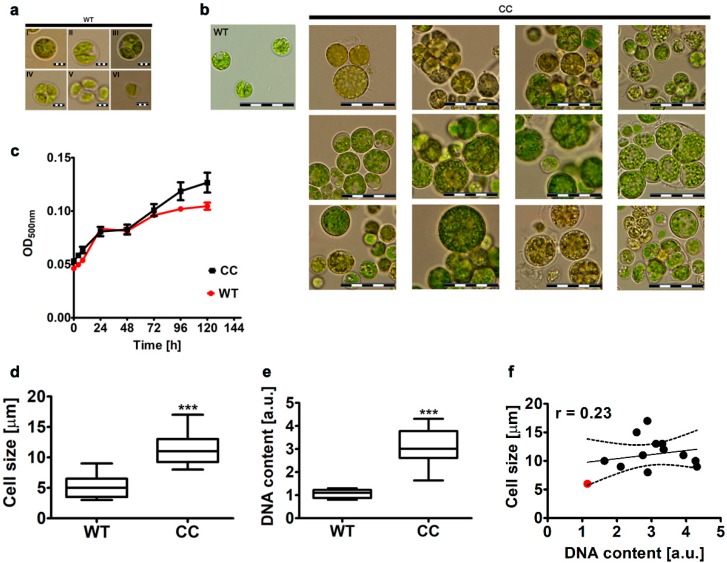
Colchicine and cytochalasin B-mediated effect on cell morphology, growth rate, cell size and DNA content in the microalga *Planktochlorella nurekis*. (**a**) Morphological features of wild type (WT) cells. Representative microphotographs are presented. Scale bars 3 μm, objective 100×. Coccoidal forms (I,II) as well as autospores in the autosporangium (III–VI) are shown. (**b**) A comparison between morphological features of WT cells and twelve CC-treated clones. Representative microphotographs are presented. Scale bars 20 μm, objective 100×. (**c**) Cell growth kinetics of WT cells (red) and CC-treated clones (black). Cell growth was monitored turbidimetrically at 500 nm in a microplate reader during a period of 120 h. Bars indicate SD, n = 3. (**d**) Morphometric analysis of cell size (diameter in µm) using a light microscopy. Box and whisker plots are shown (mean ± SD), n = 3, *** *p* < 0.001 compared to WT cells (ANOVA and Dunnett’s a posteriori test). (**e**) Fluorescence microscopy-based analysis of DNA content. DNA content of twelve CC-treated clones (CC) was compared to WT cells (WT). ImageJ software was used to analyze the nuclear DNA content. DNA content was expressed as arbitrary units [a.u.]. Box and whisker plots are shown (mean ± SD), n = 3, *** *p* < 0.001 compared to WT cells (ANOVA and Dunnett’s a posteriori test). (**f**) Correlation analysis between cell size and DNA content. Twelve CC-treated clones were considered (black dots). WT cells are denoted as a red dot. Results represent the mean from three independent experiments. The 95% confidence interval is shown. Correlation analysis of the data was performed using a Linear Correlation (Pearson r) test.

First, we have established that microalgal samples belong to the species of *Planktochlorella nurekis*. The sequencing data on the internal transcribed spacers (ITS) markers and BLAST-based search revealed that analyzed sequence has a high level of similarity (99% of identical positions, data not shown) with *Planktochlorella nurekis* sequence (accession no. emb HF677200.1). Thus, one can conclude that obtained microalgal samples are indeed *Planktochlorella nurekis* samples. Then, we have characterized the morphology of wild type strain using light microscopy (Figure 1a).

*Planktochlorella nurekis* strain was found to be of coccoidal shape with uninuclear morphology of green vegetative cells (Figure 1a I and II) and without tendency to form colonies. The vegetative cells contain single pot–shaped chloroplast with pyrenoid (Figure 1a I and II) that is usually divided into two lobes in mature cells. As autospores were revealed, one can suggest that *Planktochlorella nurekis* may reproduce asexually (Figure 1a III). The autosporangium contains usually four autospores (Figure 1a IV). The autospores may have ellipsoidal or irregular shapes (Figure 1a V and VI). Our morphological observations are in agreement with previously reported results [19]. In 2014, the microalgal strain, isolated from the Nurek reservoir in Tajikistan and initially identified as *Chlorella vulgaris* KIEG 1904, has been characterized in terms of morphological and molecular features and relocated to *Parachlorella*-clade and renamed as a new genus and species, *Planktochlorella nurekis* [19].

Second, we have considered a co-treatment with colchicine and cytochalasin B (CC) to inhibit karyokinesis and cytokinesis, respectively, and thus modulate DNA levels and cell size. Twelve CC-treated clones were selected for further analysis. As one can observe in Figure 1b, CC-treated cells are bigger than wild type (WT) cells. Growth rate of CC-treated cells was also slightly accelerated, especially after 96 h of culture (Figure 1c). We have then conducted a morphometric analysis that revealed that cell size (diameter in µm) of CC-treated cells is significantly increased compared to cell size of WT cells (*p* < 0.001, Figure 1d). A two-fold increase in cell size was noticed in CC-treated cells compared to unmodified cells (*p* < 0.001, Figure 1d). Then, we have decided to study CC-treated microalgal population more comprehensively in terms of its morpho-phenotypic features using imaging flow cytometry (Supplementary Figure S1). CC co-treatment promoted heterogeneity of microalgal population (Supplementary Figure S1). We were able to observe five distinct subpopulations denoted as R1 (cells sized ranging from 5 to 10 µm), R2 (cells sized ranging from 1 to 5 µm), R3 (cells sized ranging from 10 to 15 µm and autosporangia), R4 (cell aggregates sized over 15 µm) and R5 (dividing cells with autospores) (Supplementary Figure S1). CC treatment resulted in an increase in R1, R3, R4 and R5 fractions (Supplementary Figure S1), whereas the subpopulation of smallest cells (R2, cell size between 1 and 5 µm) was decreased compared to WT population (Supplementary Figure S1). Our new, safe non-vector method is based on the observation that plant cells are able to tolerate changes in their ploidy state that may promote new beneficial phenotypic features and even new plants e.g., new varieties of crops. Polyploidy in plants is mainly induced by the treatment with an inhibitor of tubulin polymerization, namely colchicine at the concentrations ranging from 0.5 to 2% [20]. However, such approach cannot be applied to microalga as microalgal cells are more sensitive to high concentrations of colchicine that inhibited cell proliferation and stimulated cell death. The treatment with colchicine at the concentration of 1% resulted in the inhibition of cell division and a 10-fold increase in cell volume of the microalga *Chlamydomonas eugametos* compared to untreated cells [21]. More recently, 12 h treatment with colchicine (0.1%) has been also reported to increase cell size of the cyanobacterium *Spirulina platensis* [22]. Colchicine in contrast to its ability to provide changes in genomic DNA, is not able to modify the cytoplasmic microalgal genome e.g., mitochondrial genome. That is also why our new proposed method has an advantage over previously used treatments with colchicine only as we considered a co-treatment with cytochalasin B to affect also cytokinesis. Moreover, both natural compounds were applied at relatively low non-cytotoxic concentrations, namely colchicine at the concentration of 50 µg/mL (0.005%) and cytochalasin B at the concentration of 3 µg/mL. Indeed, microalgal cells selected after CC treatment were characterized by moderate increase in growth rate compared to unmodified cells (Figure 1c). CC-treated cells were also able to produce more biomass from equal number of cells compared to unmodified cells, namely 12.35 ± 3.3 mg of DW per 10^8^ CC-treated cells versus 3.06 ± 0.6 mg of DW per 10^8^ unmodified cells. Moreover, no significant changes in cell viability were observed after 24 h stimulation with colchicine and cytochalasin B compared to untreated cells (data not shown).

Then, we decided to evaluate if CC treatment-mediated increase in cell size is also accompanied by elevated DNA levels in microalgal cells (Figure 1e). Indeed, 3-fold increase in DNA content of CC-treated cells was noticed compared to DNA content of WT cells (*p* < 0.001, Figure 1e). A positive correlation between cell size and DNA content was also noticed (r = 0.23, Figure 1f). However, this effect was not statistically significant that may be due to data dispersion and the fact that twelve CC-treated clones were considered (Figure 1f). More recently, colchicine-induced polyploidy has been also documented in the unicellular green alga *Dunaliella salina* [23]. Treatments with 0.1% and 0.5% colchicine resulted in the occurrence of poliploid cell populations of 58.26% and 74.19%, respectively, as judged by flow cytometry analysis [23]. Colchicine at the concentration of 0.5% also significantly lowered cell count and promoted cell cycle arrest in *Dunaliella salina* [23].

### 2.2. Biochemical Analyses

**Figure 2:**
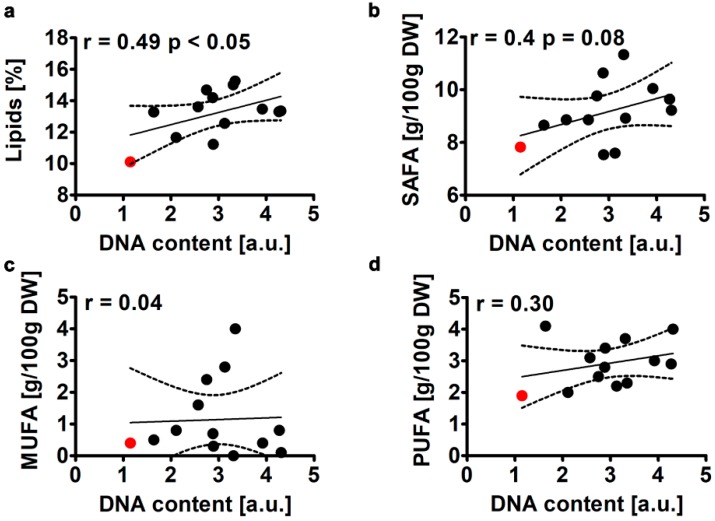
Colchicine and cytochalasin B-mediated effect on the levels of total lipids (**a**), SAFA (**b**), MUFA (**c**) and PUFA (**d**) in the microalga *Planktochlorella nurekis*. Correlation analysis between lipid content (**a**), SAFA (**b**), MUFA (**c**), PUFA (**d**) and DNA content. Twelve CC-treated clones were considered (black dots). WT cells are denoted as a red dot. Results represent the mean from three independent experiments. The levels of SAFA, MUFA and PUFA were calculated per 100 g of dry weight. The 95% confidence interval is shown. Correlation analysis of the data was performed using a Linear Correlation (Pearson r) test. DW, dry weight.

**Figure 3:**
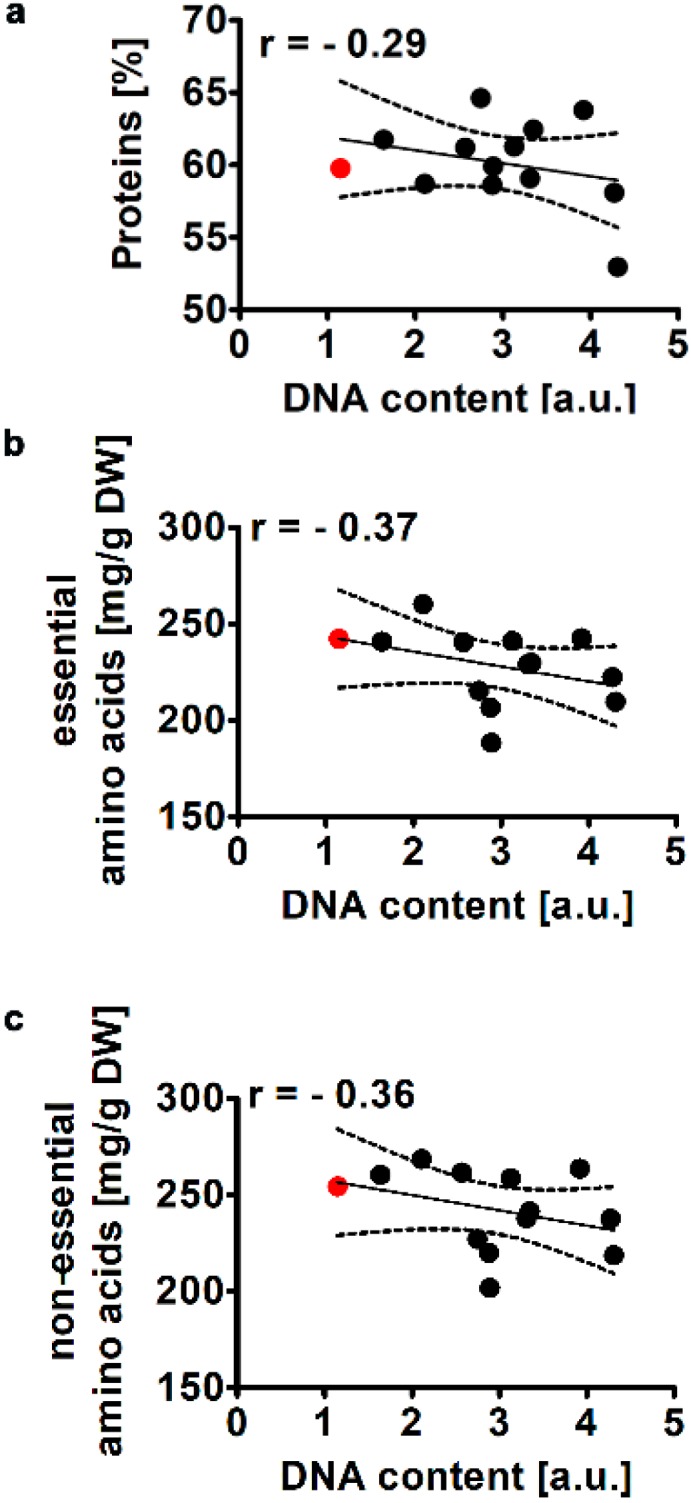
Colchicine and cytochalasin B-mediated effect on the levels of proteins (**a**), essential amino acids (**b**) and non-essential amino acids (**c**) in the microalga *Planktochlorella nurekis*. Correlation analysis between protein levels (**a**), essential amino acids (**b**), non-essential amino acids (**c**) and DNA content. Twelve CC-treated clones were considered (black dots). WT cells are denoted as a red dot. Results represent the mean from three independent experiments. The levels of amino acids [mg] were calculated per g of dry weight. The 95% confidence interval is shown. Correlation analysis of the data was performed using a Linear Correlation (Pearson r) test.

**Figure 4:**
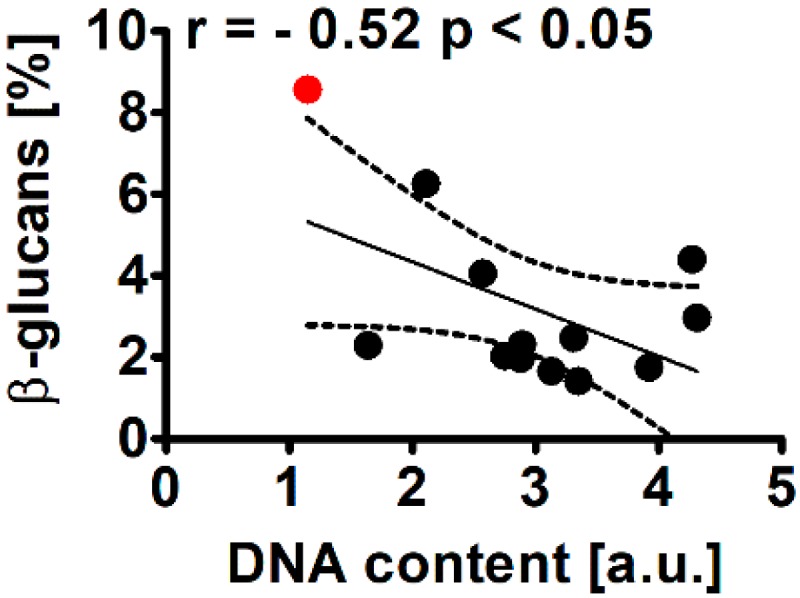
Colchicine and cytochalasin B-mediated effect on the levels of β-glucans in the microalga *Planktochlorella nurekis*. Correlation analysis between β-glucans and DNA content. Twelve CC-treated clones were considered (black dots). WT cells are denoted as a red dot. Results represent the mean from three independent experiments. The 95% confidence interval is shown. Correlation analysis of the data was performed using a Linear Correlation (Pearson r) test.

**Figure 5:**
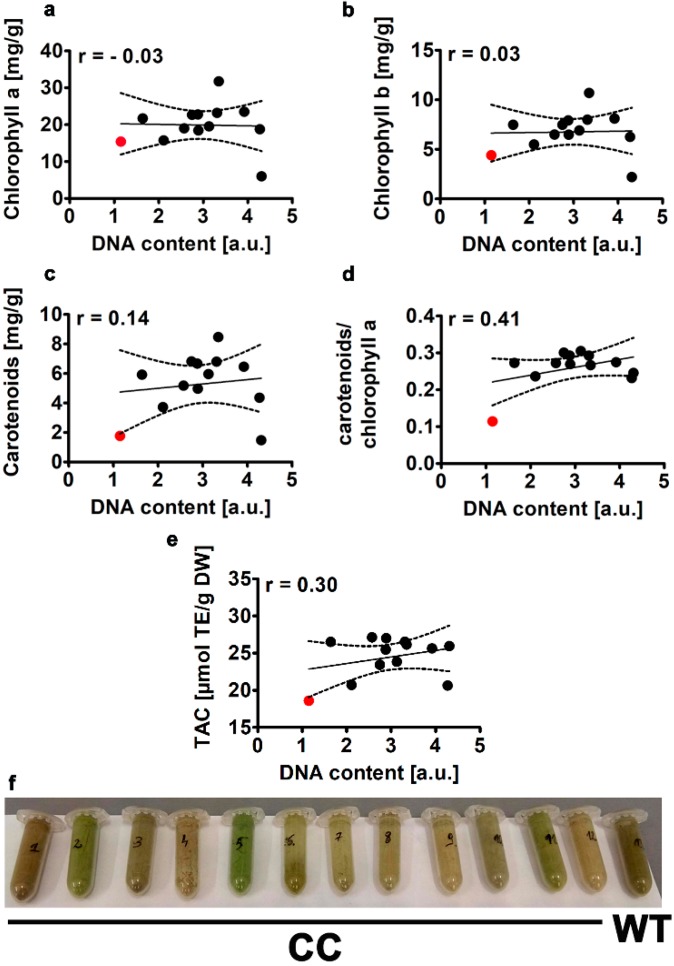
Colchicine and cytochalasin B-mediated effect on the levels of chlorophyll a (**a**), chlorophyll b (**b**), carotenoids (**c**) carotenoids to chlorophyll a ratio (**d**) and total antioxidant capacity (TAC) (**e**) in the microalga *Planktochlorella nurekis*. Correlation analysis between chlorophyll a (**a**), chlorophyll b (**b**), carotenoids (**c**) and carotenoids to chlorophyll a ratio (**d**), TAC (**e**) and DNA content. Twelve CC-treated clones were considered (black dots). WT cells are denoted as a red dot. Results represent the mean from three independent experiments. The levels of pigments and TAC were calculated per g of dry weight. The 95% confidence interval is shown. Correlation analysis of the data was performed using a Linear Correlation (Pearson r) test. The color of dry biomass of twelve CC-treated clones (1–12) and WT cells (13) is also shown (**f**). DW, dry weight.

**Figure 6:**
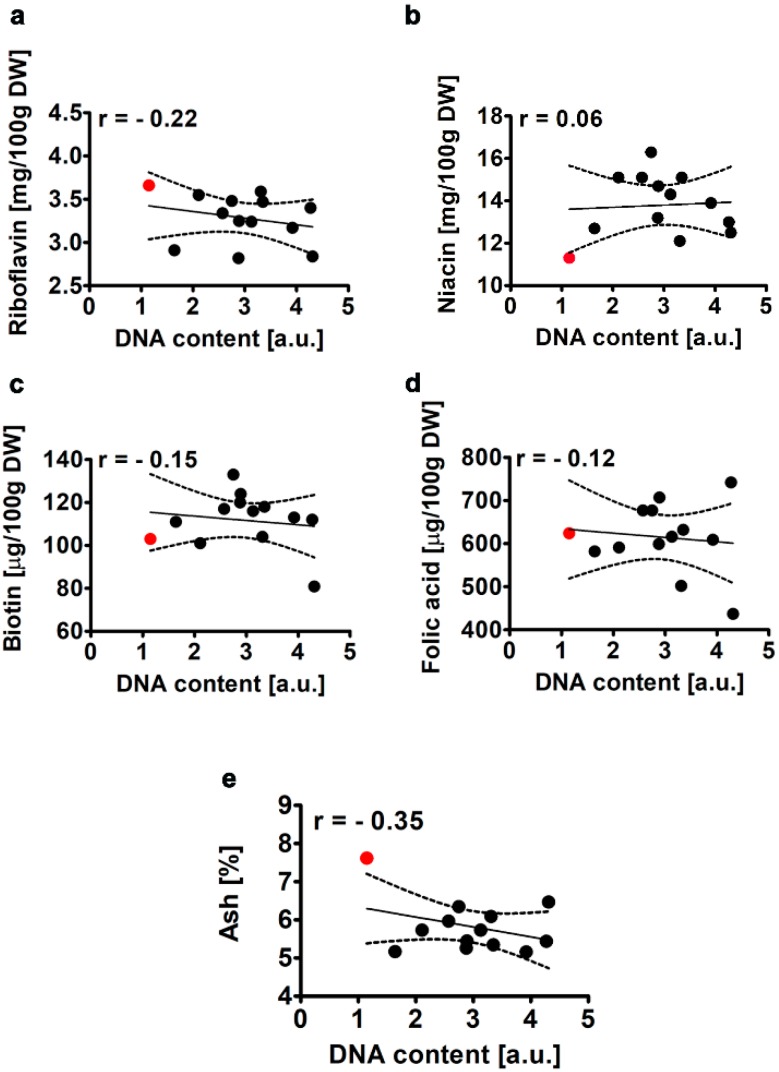
Colchicine and cytochalasin B-mediated effect on the levels of riboflavin (**a**), niacin (**b**), biotin (**c**), folic acid (**d**) and ash (**e**) in the microalga *Planktochlorella nurekis*. Correlation analysis between riboflavin (**a**), niacin (**b**), biotin (**c**), folic acid (**d**) and ash (**e**) and DNA content. Twelve CC-treated clones were considered (black dots). WT cells are denoted as a red dot. Results represent the mean from three independent experiments. The levels of vitamins were calculated per 100 g of dry weight. The 95% confidence interval is shown. Correlation analysis of the data was performed using a Linear Correlation (Pearson r) test. DW, dry weight.

Then, we have addressed the question of whether CC-induced increase in cell size and DNA content of microalgal cells may also modulate the production of biomolecules, namely lipids, fatty acids, proteins, amino acids and β-glucans, pigments, vitamins and macro- and microelements (determined as ash). Total antioxidant capacity was also assayed. Twelve CC-treated clones were considered and correlation analysis between the levels of biomolecules and DNA content was provided (Figure 2, Figure 3, Figure 4, Figure 5 and Figure 6).

#### 2.2.1. Total Lipid Content and Fatty Acid Profile

Lipid content was established to be 10% of dry weight of WT cells (a red dot, Figure 2a).

CC co-treatment resulted in an increase of the content of total fat (lipids) of about 10 to 60% in twelve modified clones considered compared to WT cells and the mean lipid content was increased of about 35% compared to WT cells (Figure 2a). Lipid content was also positively correlated with DNA content (r = 0.49, *p* < 0.05, Figure 2a). Lipid content in microalgal strains and species may vary greatly and be between 1 to 80% per dry weight [4,9,24] and values higher than 40% are suggested to be achieved under nutrient limitation [25]. For example, the content of lipids in *Chlorella vulgaris* was estimated to be between 12 to 26% of dry weight [9]. We have then considered the content of some lipid fractions and selected fatty acids, namely saturated fatty acids (SAFA) (Figure 2b), monounsaturated fatty acids (MUFA) (Figure 2c), polyunsaturated fatty acids (PUFA) (Figure 2d), lauric acid, myristic acid, pentadecanoic acid, palmitic acid, stearic acid, arachidic acid, oleic acid and linoleic acid (Supplementary Figure S2). The levels of SAFA (r = 0.4, Figure 2b) and PUFA (r = 0.3, Figure 2d) were positively correlated with DNA content, but these correlations were of no statistical significance that was due to high dispersion of data concerning all twelve CC-treated clones considered. We believe that including all twelve CC-treated clones and not selecting just the best lipid producers among them would better reflect the impact of co-treatment with colchicine and cytochalasin B on parameters analyzed and would also shed light on cellular heterogeneity. Among fatty acids considered, the strongest positive correlation between the level of fatty acid and DNA content was observed for palmitic acid (r = 0.46, Supplementary Figure S2), myristic acid (r = 0.38, Supplementary Figure S2) and linoleic acid (r = 0.29, Supplementary Figure S2). Again, these correlations were of no statistical significance (Supplementary Figure S2).

Interestingly, the oil yield [L/ha] of microalgae may be several times higher than the oil yield of oil crops, e.g., microalgae with 70% and 30% oil of total biomass may achieve 23- and 10-fold higher oil yield compared to oil palm, respectively [24]. Several approaches have been developed to enhance total lipid content and improve lipid productivity in microalgae [9,10,11]. Lipid biosynthesis may be affected by several stress-induced conditions, e.g., the manipulation of the concentrations of nutrients such as nitrogen, phosphorus, sulfur and iron, high salinity, high temperature, light intensity and alternative sources of organic carbon [8,10]. It has been reported that limitation with nitrogen, sulfur or phosphorus resulted in increased amount of lipids in *Parachlorella kessleri* [15] and nitrogen limitation was the most effective that is also observed in other microalgal species [16,26]. However, stress-mediated accumulation of lipids is typically accompanied by limited growth rate that may reduce lipid productivity as has been already documented during stress conditions in *Nannochloropsis oculata* and *Chlorella vulgaris* [16]. Thus, also genetic manipulations are considered to engineer oleaginous microalgae to both accumulate biomass and overproduce lipids [27]. For example, using a high-efficiency CRISPR–Cas9 reverse-genetics pipeline for *Nannochloropsis gaditana*, a transcriptional regulator of lipid accumulation, namely Zn(ii)_2_Cys_6_ (ZnCys) has been revealed and knockout of ZnCys transcription factor promoted partitioning of total carbon to lipids from 20% (wild type strain) to 40–55% (mutant strain) in nutrient replete conditions [27]. The growth of *Nannochloropsis gaditana* mutants lacking active ZnCys transcription factor was poor, but ZnCys-KO lines produced twice as much lipids (~5.0 g m^−2^ day^−1^) as compared to unmodified cells (~2.5 g m^−2^ day^−1^) [27]. Moreover, a targeted metabolic engineering by antisense and RNAi-based knockdown of a multifunctional lipase/phospholipase/acyltransferase (Thaps3_264297) enhanced lipid yields without compromising the growth of the diatom *Thalassiosira pseudonana* [28]. The ability to control microalgal lipid production using genetic engineering might eventually enable the commercialization of microalgal-derived biofuels [27]. However, genetic manipulations and the construction of genetically modified microalgal species containing foreign genes or markers might also raise the safety concerns when considering microalgae as a dietary supplement and a source of high-value nutrients/nutraceuticals. The ecological monitoring and research on the environment and human health impacts of genetically modified microalgae that may persist in and alter natural ecosystems are needed [17,18]. Thus, a safe non-vector approach based on colchicine and cytochalasin B co-treatment to increase DNA levels and total lipid content in *Planktochlorella nurekis* has been proposed (this study). To the best of our knowledge, this is the first report on co-treatment with colchicine and cytochalasin B-mediated enhancement of lipid content in a microalgal strain without affecting cell growth rate that is also positively correlated with DNA content. More recently, similar approach has been validated in haploid cells of the microalga *Chlamydomonas reinhardtii* treated with colcemid, a derivative of colchicine that disrupted chromosome segregation and resulted in polyploidy [29]. Two diploid clones, namely CMD ex1 and CMD ex4 were characterized by elevated yield of biomass and fatty acid methyl esters (FAME) under nitrogen starvation conditions, but under nutrient replete conditions, FAME yield of colcemid-treated cells was not increased compared to unmodified cells [29].

#### 2.2.2. Total Protein Levels and Amino Acid Profile

The total protein levels in WT *Planktochlorella nurekis* cells were established to be about 60% of dry weight (a red dot, Figure 3a).

This is in agreement with previously published data on the protein content in the genus of *Chlorella* and related genera [4,5]. The protein content in different species of *Chlorella* may vary between 11 to 68% of dry weight [1,4,5]. In general, a protein-rich *Chlorella* genus may be potentially considered as a human food and animal feed, especially as a source of essential amino acids (e.g., lysine, leucine, isoleucine, tryptophan and valine) [4,5]. In general, *Planktochlorella nurekis* amino acid profile (Supplementary Figure S3) was found to be similar to *Chlorella* amino acid profile [1]. The most abundant essential amino acids were leucine (45 mg/g of dry weight), valine (32 mg/g of dry weight) and arginine (30 mg/g of dry weight) (red dots, Supplementary Figure S3) and the most abundant non-essential amino acids were glutamic acid (56 mg/g of dry weight), aspartic acid (50 mg/g of dry weight) and alanine (45 mg/g of dry weight) in WT cells (red dots, Supplementary Figure S3). CC co-treatment resulted in a decrease in protein content (r = −0.29) (Figure 3a), the pool of essential amino acids (r = −0.37) (Figure 3b) and non-essential amino acids (r = −0.36) (Figure 3c) and almost all amino acids examined (Supplementary Figure S3). A statistically significant negative correlation was revealed between isoleucine (r = −0.51, *p* < 0.05), methionine (r = −0.63, *p* < 0.05), threonine (r = −0.54, *p* < 0.05), valine (r = −0.52, *p* < 0.05) and serine (r = −0.49, *p* < 0.05) and DNA content (Supplementary Figure S3). Our data are in agreement with previous observations that nutrient depleted condition- or stress stimuli-mediated increase in lipid content may be achieved at the expense of other components, particularly proteins [25]. A number of microalgae may modulate their biochemical profiles by the enhancement of the biosynthesis of lipids and triacylglycerols and biodegradation of proteins under nitrogen depleted condition [10].

#### 2.2.3. β-Glucans

We have then analyzed the levels of β-glucans as these glucose polymers were previously identified as high-value nutritional products, e.g., anticancer and immunostimulatory effects of β-glucans were documented [30,31]. WT cells of *Planktochlorella nurekis* were characterized by the levels of β-glucans of 8.5% of dry weight (a red dot, Figure 4).

More recently, a comprehensive screening of forty seven species of cultured microalgae for the levels of β-glucans has been performed and the levels of β-glucans in different *Chlorella* strains ranged from 6.2 to 8.9% of dry weight [32]. CC co-treatment resulted in a decrease in β-glucan content of *Planktochlorella nurekis* (Figure 4). Taking into consideration twelve CC-treated clones, 1.4- to 4.25-fold diminution in β-glucan content was observed compared to unmodified cells (Figure 4). A statistically significant negative correlation was revealed between the levels of β-glucans and DNA content (r = −0.52, *p* < 0.05, Figure 4). Moreover, CC co-treatment also promoted a decrease in the ratio of β-glucans to total glucans (data not shown). The content of β-glucans can be also modulated by nitrate starvation and irradiance [32]. Elevated irradiance resulted in an increase in the levels of β-glucans of *Scenedesmus obtusiusculus* A 189 from 6.4 to 19.5%, whereas nitrate starvation increased the content of β-glucans of *Scenedesmus obtusiusculus* A 189 from 16 to 23% and of *Scenedesmus ovalternus* SAG 52.80 from 23.3 to 36.7% [32].

#### 2.2.4. Pigments and Total Antioxidant Capacity

At least three classes of natural pigments can be found in microalgae and cyanobacteria, namely phycobilins, chlorophylls and carotenoids [9]. Besides their important roles in the process of photosynthesis and pigmentation, microalgal pigments may promote a number of beneficial biological effects such as antioxidant, anticancer, anti-inflammatory, anti-obesity, anti-angiogenic and neuroprotective effects and thus may be considered as high-value nutraceuticals and pharmaceuticals [9,33]. The chlorophyll yield may be improved by light intensity, culture agitation, and changes in temperature and nutrient availability [34]. For example, optimum light-dark cycle, decreased light intensity, red light, high availability of phosphorus and increased temperature may promote chlorophyll accumulation in microalgae [34]. However, such modulations may be also species-dependent [34]. We have analyzed the effect of CC co-treatment on the levels of chlorophyll a and b as well as the content of carotenoids and the ratio of carotenoids to chlorophyll a (Figure 5).

The levels of chlorophyll a (15 mg/g, 1.5%) and chlorophyll b (4.5 mg/g, 0.45%) of WT cells (red dots, Figure 5a,b) were comparable to those of previously established in microalgal species as chlorophyll levels may vary between 0.5 to 1% of dry weight in microalgae [9]. We have also established the total carotenoid content to be about 2 mg per g of dry weight (a red dot, Figure 5c) but we did not analyze further the levels of particular carotenoids, e.g., β-carotene. Our data are in agreement with previously published results on the levels of total carotenoids in different *Chlorella* samples as total carotenoid content may very between 0.25 to 3.04 mg per g of dry weight in *Chlorella* sp. [35]. The authors also showed that carotenoid content was positively correlated with total antioxidant capacity (TAC) of *Chlorella* strains [35]. The main carotenoids of *Chlorella* are: β-carotene, lycopene, astaxanthin, zeaxanthin, violaxanthin, and lutein [4]. Some keto-carotenoids such as canthaxanthin and astaxanthin can be also found in selected *Chlorella* species [4]. CC co-treatment did not affect the levels of both chlorophyll a and b (Figure 5a,b), however the levels of carotenoids and the ratio of carotenoids to chlorophyll a were slightly to moderately increased compared to unmodified cells (Figure 5c,d). A positive correlation between the ratio of carotenoids to chlorophyll a and DNA content was observed (r = 0.41, Figure 5d). Stress conditions e.g., nutrient limitation may also affect the content of carotenoids in microalgae [36,37,38]. Nitrogen stress promoted the biosynthesis of carotenoids but excessive nitrogen stress resulted in reduced proliferative and photosynthetic activity in *Chlorella vulgaris* [36]. It has been reported that nutrient depleted conditions enhanced the biosynthesis of all-trans-astaxanthin from 0.03 to 0.11 mg/g of dry weight, all-trans-lutein from 2.35 to 4.18 mg/g of dry weight and all-trans-canthaxanthin from 0.27 to 1.15 mg/g of dry weight in the green microalga *Coelastrum* sp. TISTR 9501RE [37]. The authors have suggested that modulating the levels of microalgal carotenoids with strong antioxidant activity may be beneficial in terms of their applications as high-value nutraceuticals and pharmaceuticals [37]. The antioxidant activity of *Chlorella vulgaris* ethanolic extract was reported to be more potent than the antioxidant activity of ethanolic extracts of *Porphyridium cruentum*, *Phaeodactylum tricornutum* and synthetic antioxidants, namely BHA (butylated hydroxyanisole) and BHT (butylated hydroxytoluene) [39]. Thus, this suggests the usefulness of microalgal preparations as dietary supplements and in the preservation of foods [39]. TAC values of unmodified samples of *Planktochlorella nurekis* were comparable to TAC values of different *Chlorella* sp. samples [35]. CC co-treatment-mediated increase in carotenoids (Figure 5d) was also accompanied by elevated TAC (Figure 5e). CC co-treatment resulted in enhanced TAC from 20 to 50% in twelve CC-treated clones considered compared to unmodified cells (Figure 5e). This diversity may also reflect different response of clones to CC-mediated changes in pigmentation (Figure 5f). A positive correlation between TAC and DNA content was also observed (r = 0.3, Figure 5e). Colcemid-associated changes in the ploidy of the microalga *Chlamydomonas reinhardtii* were also accompanied by cold and oxidative stress resistance [29]. It has been also reported that 0.025% colchicine treatment resulted in the accumulation of phycocyanin, a pigment with antioxidant properties in the cyanobacterium *Spirulina platensis* [22]. However, the authors did not analyze if colchicine-mediated increase in phycocyanin content may promote oxidative stress resistance or related adaptive responses.

#### 2.2.5. Selected B Vitamins and Ash Content

As *Chlorella* may also contain other beneficial components such as vitamins (e.g., B-complex vitamins) and macro- and microelements (e.g., potassium, sodium, magnesium, iron and calcium) [1], we decided then to analyze the effect of CC co-treatment on the levels of selected vitamins (B2, B3, B7 and B9) (Figure 6a–d) as well as the content of ash (Figure 6e).

The established contents of riboflavin, niacin, biotin and folic acid in *Planktochlorella nurekis* WT cells (red dots, Figure 6a–d) were comparable to those obtained for *Chlorella* strains [1,40]. A strategy to augment the levels of niacin may be beneficial in terms of the applications of microalgal preparations as dietary supplements to improve e.g., the brain functions [41]. There are a number of biological processes that are dependent on niacin derived nucleotides such as nicotinamide adenine dinucleotide (NAD) and NAD phosphate (NADP), namely energy production, oxidative reactions, antioxidant protection, DNA metabolism and repair, cellular signalling events and the conversion of folate to its tetrahydrofolate derivative [41]. However, CC co-treatment did not result in significant increase in the levels of niacin (Figure 6b). The levels of other B vitamins were rather decreased after CC co-treatment (Figure 6a,c,d). No clear correlation between the levels of B vitamins and CC co-treatment was revealed (Figure 6a–d). CC co-treatment also diminished the levels of ash that may reflect decreased content of macro- and microelements (Figure 6e). However, this correlation was not statistically significant.

## 3. Materials and Methods

### 3.1. Species Identification and Culture Conditions

The microalga samples were provided by Bioorganic Technologies sp. z o.o. (Sielec, Poland). Species identification was based on DNA sequencing. Briefly, nucleic acids were extracted from the algae using 5% Chelex-100 as described elsewhere [42]. The samples were boiled at 100 °C for 15 min, centrifuged (16 000× *g*, 2 min) and the amount of obtained DNA was quantified using a NanoDrop 2000 Spectrophotometer (Thermo Fisher Scientific, Warsaw, Poland). The sequences of ITS regions were obtained by PCR amplification using Mastercycler gradient thermal cycler (Eppendorf, Warsaw, Poland), and a pair of primers (P1: 5′-ACTCCGCCGGCACCTTATGAG-3′, P2: 5′-CCGGTTCGCTCGCCGTTACTA-3′) [43]. Each reaction mixture contained 300 ng of DNA, 1× PCR Mix with 0.5 mM dNTP (dATP, dCTP, dGTP, dTTP), 2.5 U recombinant DNA polymerase, and 0.1 μM of forward and reverse primers. The ITS region was amplified using the following conditions: an initial denaturation at 95 °C for 2 min, 35 cycles at 94 °C for 30 s (denaturation), 66 °C for 30 s (primer annealing), 72 °C for 2 min (primer extension) and a final elongation step at 72 °C for 10 min. PCR products were separated using 1% 1× TBE agarose gel electrophoresis, stained with ethidium bromide and visualized under UV light (GBox, Thermo Fisher Scientific, Warsaw, Poland). The molecular weight of the DNA fragments were determined using a perfect weight marker 1 kb DNA Ladder (EURx, Gdansk, Poland) and then purified by gel extraction kit (Gel Purification GPB Mini Kit, GenoPlast, Rokocin, Poland). DNA samples were sequenced in the Laboratory of DNA Sequencing and Oligonucleotides Synthesis, Institute of Biochemistry and Biophysics of the Polish Academy of Sciences, Warsaw, Poland. The obtained sequences were aligned and compared to the nucleotide sequences in GeneBank database of the National Center for Biotechnology Information (NCBI) by using BLAST. The microalga samples were identified as *Planktochlorella nurekis* species.

The *Planktochlorella nurekis* strains were grown in 120 L photobioreactors in liquid medium containing the following macro- and microelements: KNO_3_ (5 g/L), MgSO_4_ (2.5 g/L), KH_2_PO_4_ (1.25 g/L), (NH_4_)_2_SO_4_ (0.6 g/L), FeSO_4_x7H_2_O (3.7 mg/L), MnCl_2_x4H_2_O (2.2 mg/L), H_3_BO_3_ (1.5 mg/L), CoCl_2_x6H_2_O (15 µg/L), ZnSO_4_x7H_2_O (0.25 mg/L), CuSO_4_x5H_2_O (0.25 mg/L), NiSO_4_x6H_2_O (0.5 mg/L), NH_4_VO_3_ (23 µg/L), MoO_3_ (18 µg/L), Na_2_WO_4_x2H_2_O (0.16 mg/L), KI (0.05 mg/L) and EDTA (0.04 g/L) (Chempur, Piekary Slaskie, Poland), pH 5.85–5.95 at 30 ± 1.0 °C under continuous artificial LED red light (645 nm) and blue light (460 nm) (ratio 3:1) and 16,000 LUX. Three independent cultures were considered.

### 3.2. Co-Treatment with Colchicine and Cytochalasin B

For long-term selection, microalgal cells (1 × 10^6^/mL, 6-well plate, a total volume of 2 mL) were treated with colchicine (50 μg/mL, Sigma-Aldrich, Poznan, Poland) and cytochalasin B (3 μg/mL, Sigma-Aldrich, Poznan, Poland) (CC treatment) at 30 °C for 24 h with shaking (500 rpm/min) and continuous red and blue light (P.426103). Then, the cells were selected without colchicine and cytochalasin B for further sub-culturing in 6-well plates for 96 h and 250 mL flasks for 10 days. When the cell culture reached 5 × 10^5^ cells/mL, the cells (1–3 × 10^4^/mL, a total volume of 250 mL) were transferred to 2.5 L photobioreactors and grown in cell culture medium with CO_2_ flow (8%), continuous red and blue light at 30 °C for 7 days. Typically, the cell density of 6 × 10^6^ cells/mL and 2 g of wet biomass per liter of culture were achieved at the end of the culture. To ensure that 24 h co-treatment with colchicine and cytochalasin B resulted in stable phenotype of increased cell size and DNA content compared to untreated cells, cells were additionally cultured without colchicine and cytochalasin B for 30 days and DNA levels were compared (time 0 and after 30 days) and cells were then propagated in 120 L bioreactors. During all selection steps, the cells were monitored using a light microscope (objectives 40× and 100×). The cell number and cell viability were monitored using a TC10™ automated cell counter and trypan blue staining (BioRad, Warsaw, Poland) and measurements of optical density (OD) using a TECAN microplate reader at 500 nm. After CC treatment and cell selection in 2.5 L bioreactors, twelve clones were considered for further analysis based on the most potent proliferative activity as judged by OD values (OD measurements at 500 nm using a microplate reader). Wet biomass was obtained after centrifugation of the cell culture (3000× *g*, 5 min) and dry mass was obtained using thin layer drying method using a moisture analyzer at 42 °C for 24 h.

### 3.3. Imaging Flow Cytometry-Based Analysis of Cell Size and Cell Aggregates

The size of microalgal cells was analyzed using an Olympus BX61 differential interference contrast microscope equipped with a DP72 CCD camera and Olympus CellF (Olympus, Warsaw, Poland). Moreover, the subpopulations of cells in terms of their cell size and formation of cell aggregates were investigated using Amnis^®^ FlowSight^®^ imaging flow cytometer and IDEAS software version 6.2.187.0 (Merck Millipore, Warsaw, Poland). 10,000 events were analyzed for each sample triplicate using two channels (bright field), namely Ch01 (435–480 nm) and Ch09 (570–595 nm) with the 488 nm and 642 nm lasers. Five subpopulations of cells were considered, namely single cells sized 5–10 µm (R1), single cells sized 1–5 µm (R2), large single cells sized 10–15 µm and autosporangia (R3), cell aggregates sized over 15 µm (R4) and dividing cells with autospores (R5). Representative dot plots and cell images are presented.

### 3.4. DNA Content Analysis

DNA content analysis was performed as comprehensively described elsewhere [44] with minor modifications, namely pigments (chlorophylls and carotenoids) were extracted by adding a mixture of acetone, methanol and water (80:15:5) (*v*/*v*/*v*) in a total volume of 1 mL to 50 mg of cell pellet (wet biomass). Cell suspensions were mixed and centrifuged (9000 rpm, 10 min). These steps were repeated three times.

### 3.5. Biochemical Analyses

#### 3.5.1. Protein Content and Amino Acid Profile

The protein levels were determined according to Kjeldahl method in laboratory accredited according to ISO/IEC 17025:2005 (JARS, Poland, certificate of the Polish Center for Accreditation no AB 1095, test procedure PB-14/LF). This method is based on the mineralization of the sample using concentrated sulphuric acid (H_2_SO_4_) and liberation of reduced nitrogen as ammonium sulphate. After alkalization of the mixture, the produced ammonia was distilled off with water vapor and bound in a boric acid solution. The distillate was titrated with a standard solution of hydrochloric acid. Protein content was calculated by multiplying the total Kjeldahl nitrogen by nitrogen-to-protein conversion factor of 5.95 that is recommended for *Chlorella* sp. [45].

Amino acids were determined in laboratory accredited according to ISO/IEC 17025:2005 (National Feed Laboratory, Poland, certificate of the Polish Center for Accreditation no AB 856).

Tryptophan was determined by high performance liquid chromatography with fluorescence detection (HPLC-FLD) method (Commission Regulation (EC) No. 152/2009 of January 27, 2009, Annex III G). The method for the determination of total tryptophan consisted of hydrolysis in alkaline medium using a saturated solution of barium hydroxide and heating the sample at 110 °C for 20 h. After hydrolysis, an internal standard was added, namely ISTD α-methyl-tryptophan. The following conditions were considered: column C18 (125 mm × 4 mm, 3 μm packing); column temperature: room temperature; isocratic elution; mobile phase: 3.00 g acetic acid + 900 mL water + 50.0 mL solution of 1,1,1-trichloro-2-methyl-2-propanol in methanol (1 g/100 mL) adjusted pH to 5.00 using ethanolamine and made up to 1 000 mL with water; flow rate: 1 mL/min; total run time: 34 min; detection wavelength: excitation: 280 nm, emission: 356 nm; injection volume: 20 μL. The other amino acids were determined using ultra-high performance liquid chromatography with spectrophotometric detection (UPLC-UV) method (test procedure PB 59 KLP). The method consisted of hydrolysis with 6N hydrochloric acid solution and the extraction of hydrolysis products with water. The obtained extract was subjected to pre-column derivatization using 6-aminoquinoline-*N*-hydroxysuccinimidyl carbamate (AQC) as the reactant followed by ultra-performance liquid chromatography (UPLC) analysis with spectrophotometric detection (PDA) at 260 nm and using the AccQ* Tag column Ultra, C-18, 2.1 × 100 mm, 1.7 μm.

#### 3.5.2. Total Lipid Content and Fatty Acid Profile

Total fat (lipids) was determined in laboratory accredited according to ISO/IEC 17025:2005 (JARS, Poland, certificate of the Polish Center for Accreditation no AB 1095, test procedure the PB-69/LF). The method is based on hydrolysis of the sample with a hydrochloric acid solution to release fat from protein-fat and sugar-fat complexes, then separating the fat from the hydrolyzate by filtration through a filter paper, drying the filter and extracting the fat in a Soxhlet apparatus and determining the weight of fat content in the sample.

For determination of SAFA, MUFA, PUFA and selected fatty acids, Laboratory Analytical Procedure (LAP) was used [46]. Briefly, 10 mg of algae (dry weight) was weighed into a 2 mL chromatographic vial. 25 μL of internal standard tripentadecanoin (1000 μg/mL), 200 μL of dichloromethane: methanol (2:1, *v*/*v*) and 300 μL of 0.6 M HCl in methanol were added to the sample. The vials were sealed (PTFE caps) and the content of the vial was mixed and placed in a laboratory oven (S-40, Alpina, Poland) heated to 85 °C ± 3 °C for 1 h. The vials were then cooled to room temperature. After cooling, 1 mL of petroleum ether was added to a vial and the content was mixed for 1 min and allowed to separate for 1 h. 100 μL of the upper phase was transferred to a 2 mL chromatographic vial and 400 μL of petroleum ether was added. The analysis was carried out using a gas chromatograph (Agilent Technologies, model 7890A, Palo Alto, CA, USA) with a mass detector (Agilent Technologies, model 7000, Palo Alto, CA, USA) in full scan mode. The following parameters were considered: ions from 50 *m*/*z* (mass to charge ratio) to 400 *m*/*z* were monitored, source temperature—230° C, ionization type—electron (EI), temperature program: 40–260 °C, column HP-5 MS (Ultra Inert/ 30 m × 0.25 mm I.D. × 0.25-μm). For identification and quantification of FAs external standard was used (37 Supelco component FAME MIX CRM47885, Merck KGaA, Germany). The linearity was determined on the basis of six point calibration curves (R^2^ from 0.925 to 0.999).

#### 3.5.3. β-Glucan Levels

The analysis of β-glucan content was performed according to manufacturer’s instructions (β-Glucan Assay Kit, K-BGLU, Megazyme, USA) and [30,32] with minor modifications, namely the pigments from the samples were removed by incubation of dry biomass with 96% ethanol at 70 °C for 2 h and centrifugation and drying the pellets at 60 °C for 24 h. Total content of glucans as well as the fractions of α-glucans and β-glucans were considered.

#### 3.5.4. The Content of Chlorophylls and Carotenoids

The content of pigments (chlorophylls, carotenoids) was assayed in dry biomass (50 mg) after extraction using 1 mL of 80% acetone (*v*/*v*). The samples were then mixed (30 min), sonicated in an ultrasonic bath (5 min), incubated at 40 °C for 30 min with shaking (400 rpm) and centrifuged (3000 rpm, 5 min). The obtained supernatant was dissolved using 80% acetone (*v*/*v*) to give an absorbance ≤ 1. The measurements were performed using quartz cuvettes in a spectrophotometer Evolution™ 300 UV-Vis (Thermo Fisher Scientific, USA) at λ = 646.8 nm, 663.2 nm and 470 nm against 80% acetone as a blank. The samples were protected from light during the analysis. The following formula was used to calculate the content of chlorophyll a, chlorophyll b and carotenoids (µg/mL) using 80% acetone (*v*/*v*) as a solvent [47]:

$$C_{a}=12.25A_{663.2}\;-\;2.79A_{646.8}$$

$$C_{b}\;=21.5A_{646.8}\;-\;5.1A_{663.2}$$

$$\mathrm{Cx}+c\;=\;\left(1000A_{470}\;-\;1.82C_{a}\;-85.02C_{b}\right)/198$$

where A is an absorbance, C_a_ is a concentration of chlorophyll a, C_b_ is a concentration of chlorophyll b and Cx + c is a concentration of total carotenoids.

#### 3.5.5. Total Antioxidant Capacity (TAC)

TAC of microalgal ethanolic extracts was evaluated using DPPH assay [48]. Briefly, 35 µL of microalgal sample (or trolox) were mixed with 115 µL of DPPH (2,2-di(4-tert-octylphenyl)-1-picrylhydrazyl) solution (a final concentration of 0.1 mM) and incubated for 15 min in the dark at room temperature. The absorbance was then measured using a microplate reader at 515 nm against a reagent blank (ethanol). Calculations were made on the basis of standard curve obtained for a trolox solution. Total antioxidant capacity is expressed as µmol trolox equivalents (TE) per g of dry weight.

#### 3.5.6. The Content of B Vitamins

Vitamins were determined according to JARS, Poland, certificate of the Polish Center for Accreditation no AB 1095, test procedure the USP 34, method 441 (mod.), microb. Act. Riboflavin was determined using HPLC method (VDLUFA Bd. III, 13.9.1). The sample was extracted using a methanolic solution with Titriplex V. The extract was separated by HPLC on a reversed phase column. The quantification of vitamin B2 was performed using UV-detection at 268 nm (Limit of Quantification (LOQ): 0.05 mg/100 g). Niacin was determined according to microbiological activity USP 30, 2007, Meth. 441 (with test organism: *Lactobacillus plantarum* ATCC 8014). The test organism was provided with a liquid nutrient medium that contained an adequate quantity of all required growth nutrients except niacin that is essential for its growth. Addition of sample extract with hydrochloric acid or standard solution, containing niacin enabled growth of the organism. After sample extraction, graded amounts of sample or standard solutions were given to tubes with nutrient medium and were inoculated with the specific test organism. After incubation, the response was measured turbidimetrically and compared to that of calibration solutions with known concentrations (LOQ: 0.04 mg/100 g). Biotin was determined according to microbiological activity U.S. Pharmacopeia, USP 21, 3rd supplement, 1986, Method 88 (with test organism: *Lactobacillus plantarum*, ATCC 8014). The test organism was provided with a liquid nutrient medium that contained an adequate quantity of all required growth nutrients except biotin that is essential for its growth. Addition of sample extract with sodium hydroxide or standard solution, containing biotin enabled growth of the organism. After sample extraction, graded amounts of sample or standard solutions were given to tubes with nutrient medium and were inoculated with the specific test organism. After incubation, the response was measured turbidimetrically and compared to that of calibration solutions with known concentrations (LOQ: 1 µg/100 g). Folic acid was determined according to microbiological activity modified EN 14131 (with test organism *Lactobacillus casei subsp. rhamnosus*, ATCC 7469). The test organism was provided with a liquid nutrient medium that contained an adequate quantity of all required growth nutrients except folic acid that is essential for its growth. Addition of sample extract or standard solution containing folic acid enabled growth of the organism. After sample extraction, graded amounts of sample or standard solutions were given to tubes with nutrient medium and inoculated with the specific test organism. After incubation, the response was measured turbidimetrically and compared to that of calibration solutions with known concentrations (LOQ: 5 µg/100 g).

#### 3.5.7. Ash Content

For ash content analysis, the crucibles were pre-conditioned in a muffle furnace at 575 °C for 20 h, cooled to a temperature of about 100 °C and transferred to a desiccator. After reaching room temperature, the crucibles were weighed on the analytical balance with an accuracy of 0.1 mg. Samples of microalgae were weighed into the crucibles (100 mg ± 5 mg). Samples were burned in a muffle furnace at 575 °C for 6 h. After cooling to a temperature of about 100 °C, the crucibles were transferred to the desiccator and after reaching room temperature, the crucibles and ashes were weighed. Ash content was calculated from the formula:

$$\%\;\mathrm{ash}\;=\;\left(m_{3}\;-\;m_{1}\right)/m_{2}\;\left[\%\;\mathrm{weight}\right]$$

where m_1_ is a crucible mass (g), m_2_ is a mass of algae (g) and m_3_ is a mass of the crucible with the residue after burning (g).

### 3.6. Statistical Analysis

The mean values ± SD were calculated on the basis of at least three independent experiments. Box and whisker plots were also considered. Statistical significance was evaluated using GraphPad Prism 5 using one-way ANOVA and Dunnett’s test. Correlation analysis of the data was performed using a Linear Correlation (Pearson r) test. A *p*-value < 0.05 was considered as a statistically significant.

## 4. Conclusions

We strongly believe that established protocol of a co-treatment with colchicine and cytochalasin B (CC) to manipulate DNA levels and modulate functional bio-components may be widely used to obtain microalgal cells of different species with improved biochemical features that may have potential applications in food and renewable energy industries. CC-modified microalgal cells may be considered as a more attractive source of dietary supplements, nutraceuticals and as a biofuel feedstock compared to untreated cells. More studies involving other microalgal species are needed to confirm such assumptions.

## Supplementary Materials

The following are available online, Figure S1: Colchicine and cytochalasin B-mediated effect on cell size, population heterogeneity and DNA content in the microalga *Planktochlorella nurekis*. (a,b) Cell morphology (here cell size) was analyzed using bright field (BF, Ch01, 435–480 nm; Ch09, 570–595 nm) using Amnis^®^ FlowSight^®^ imaging flow cytometer and IDEAS software (Merck Millipore). Five subpopulations of cells were considered, namely R1 (cells sized ranging from 5 to 10 µm), R2 (cells sized ranging from 1 to 5 µm), R3 (cells sized ranging from 10 to 15 µm and autosporangia), R4 (cell aggregates sized over 15 µm) and R5 (dividing cells with autospores) [%]. Representative dot plots and cell images are presented. Other channels are also shown, namely Ch02 (480–560 nm), Ch03 (560–595 nm), Ch04 (595–642 nm), Ch05 (642–745 nm) and Ch06 (745–780 nm). The auto-fluorescence reflects the content of various pigments. (c) Fluorescence microscopy-based analysis of DNA content. Cells were analyzed using an Olympus BX61 fluorescence microscope equipped with a DP72 CCD camera and Olympus CellF software (Olympus). For DNA visualization, the slides were counterstained with a drop of mounting medium containing 4′,6′-diamino-2-phenylindole (DAPI) (blue). DNA content was expressed as arbitrary units [a.u.]. Representative microphotographs and data distribution (histograms) are shown. Figure S2: Colchicine and cytochalasin B-mediated effect on lauric acid, myristic acid, pentadecanoic acid, palmitic acid, stearic acid, arachidic acid, oleic acid and linoleic acid in the microalga *Planktochlorella nurekis*. Correlation analysis between lauric acid, myristic acid, pentadecanoic acid, palmitic acid, stearic acid, arachidic acid, oleic acid and linoleic acid and DNA content. Twelve CC-treated clones were considered (black dots). WT cells are denoted as a red dot. Results represent the mean from three independent experiments. The levels of selected fatty acids were calculated per 100 g of dry weight. The 95% confidence interval is shown. Correlation analysis of the data was performed using a Linear Correlation (Pearson r) test. DW, dry weight. Figure S3: Colchicine and cytochalasin B-mediated effect on the levels of essential amino acids (top) and non-essential amino acids (bottom) in the microalga *Planktochlorella nurekis*. Correlation analysis between essential amino acids, non-essential amino acids and DNA content. Twelve CC-treated clones were considered (black dots). WT cells are denoted as a red dot. Results represent the mean from three independent experiments. The levels of amino acids [mg] were calculated per g of dry weight. The 95% confidence interval is shown. Correlation analysis of the data was performed using a Linear Correlation (Pearson r) test.
