# Supplementary material for: A Non-Vector Approach to Increase Lipid Levels in the Microalga Planktochlorella nurekis
Source: Molecules. 2020 Jan 9;25(2):270. doi: 10.3390/molecules25020270 (PMC7024195; doi:10.3390/molecules25020270)
Supplement: Supplementary file 1 [file molecules-25-00270-s001.pdf]

Supplementary Figures

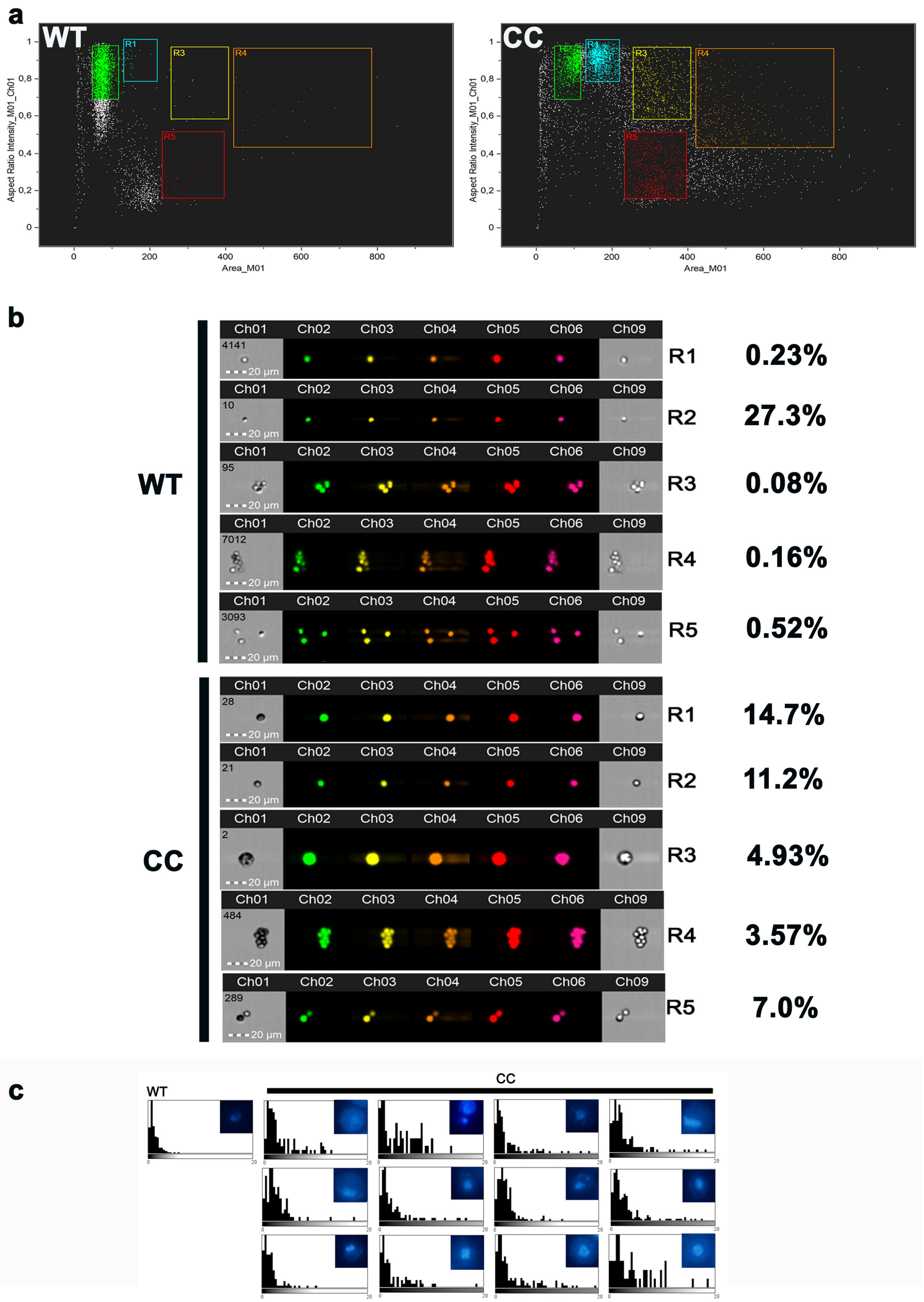

Supplementary Figure 1.

Colchicine and cytochalasin B-mediated effect on cell size, population heterogeneity and DNA content in the microalga *Planktochlorella nurekis*. (**a**, **b**) Cell morphology (here cell size) was analyzed using bright field (BF, Ch01, 435-480 nm; Ch09, 570-595 nm) using Amnis® FlowSight® imaging flow cytometer and IDEAS software (Merck Millipore). Five subpopulations of cells were considered, namely R1 (cells sized ranging from 5 to 10 µm), R2 (cells sized ranging from 1 to 5 µm), R3 (cells sized ranging from 10 to 15 µm and autosporangia), R4 (cell aggregates sized over 15 µm) and R5 (dividing cells with autospores) [%]. Representative dot plots and cell images are presented. Other channels are also shown, namely Ch02 (480-560 nm), Ch03 (560-595 nm), Ch04 (595-642 nm), Ch05 (642-745 nm) and Ch06 (745-780 nm). The auto-fluorescence reflects the content of various pigments. (**c**) Fluorescence microscopy-based analysis of DNA content. Cells were analyzed using an Olympus BX61 fluorescence microscope equipped with a DP72 CCD camera and Olympus CellF software (Olympus). For DNA visualization, the slides were counterstained with a drop of mounting medium containing 4′,6′-diamino-2-phenylindole (DAPI) (blue). DNA content was expressed as arbitrary units [a.u.]. Representative microphotographs and data distribution (histograms) are shown.

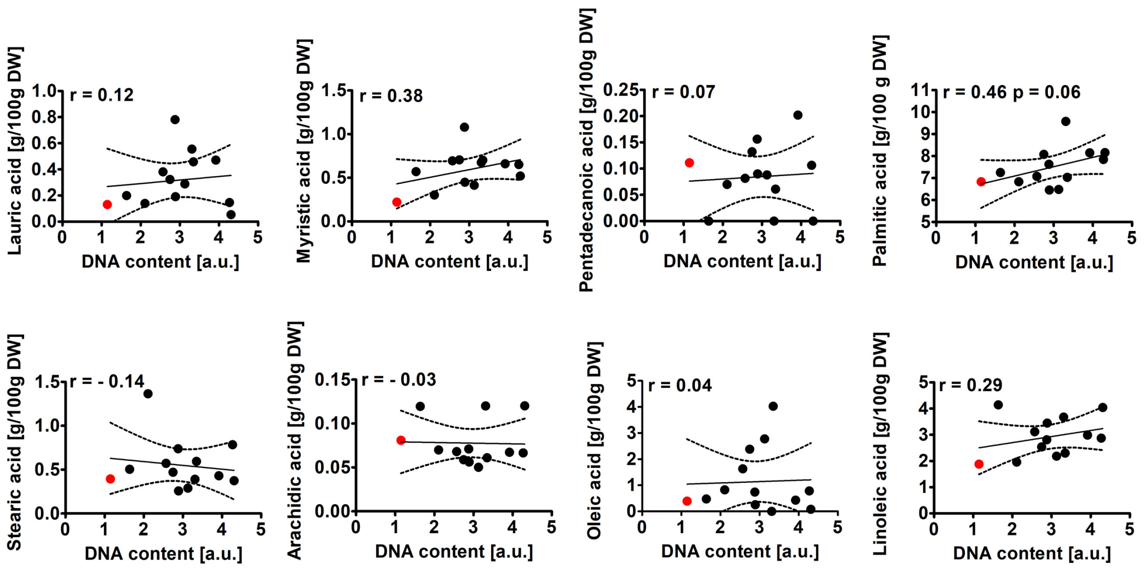

Supplementary Figure 2.

Colchicine and cytochalasin B-mediated effect on lauric acid, myristic acid, pentadecanoic acid, palmitic acid, stearic acid, arachidic acid, oleic acid and linoleic acid in the microalga *Planktochlorella nurekis*. Correlation analysis between lauric acid, myristic acid, pentadecanoic acid, palmitic acid, stearic acid, arachidic acid, oleic acid and linoleic acid and DNA content. Twelve CC-treated clones were considered (black dots). WT cells are denoted as a red dot. Results represent the mean from three independent experiments. The levels of selected fatty acids were calculated per 100 g of dry weight. The 95% confidence interval is shown. Correlation analysis of the data was performed using a Linear Correlation (Pearson r) test. DW, dry weight.

essential amino acids

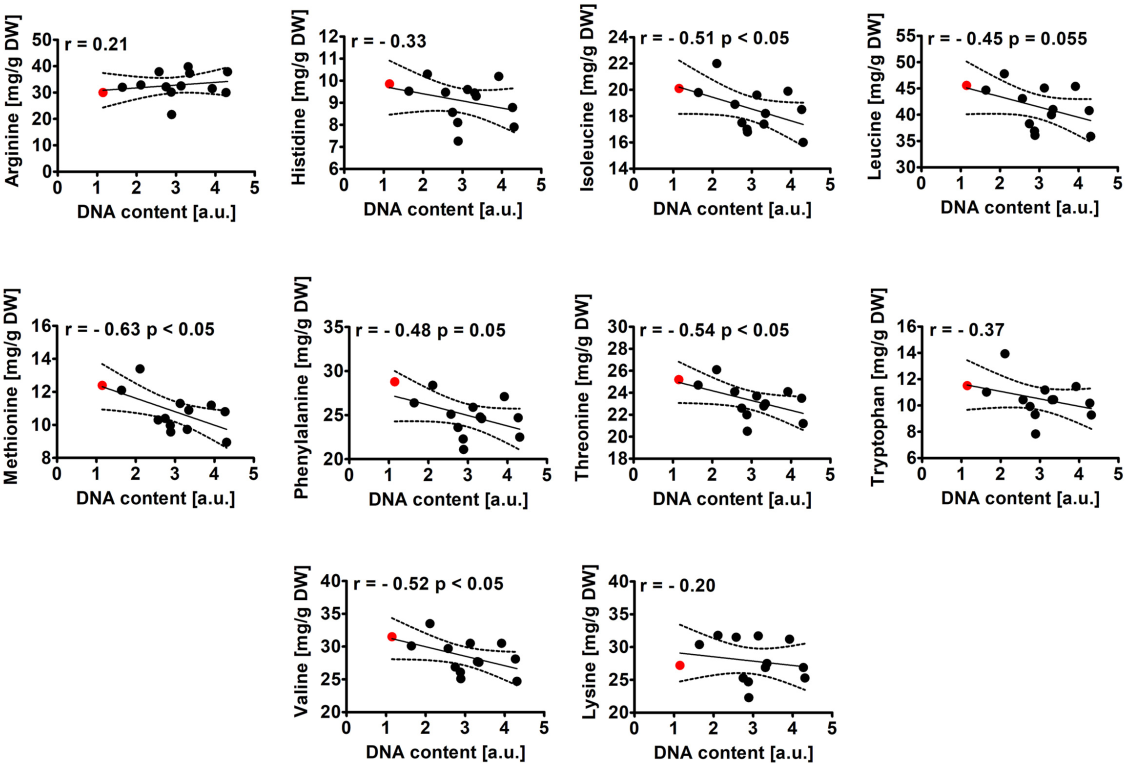

non-essential amino acids

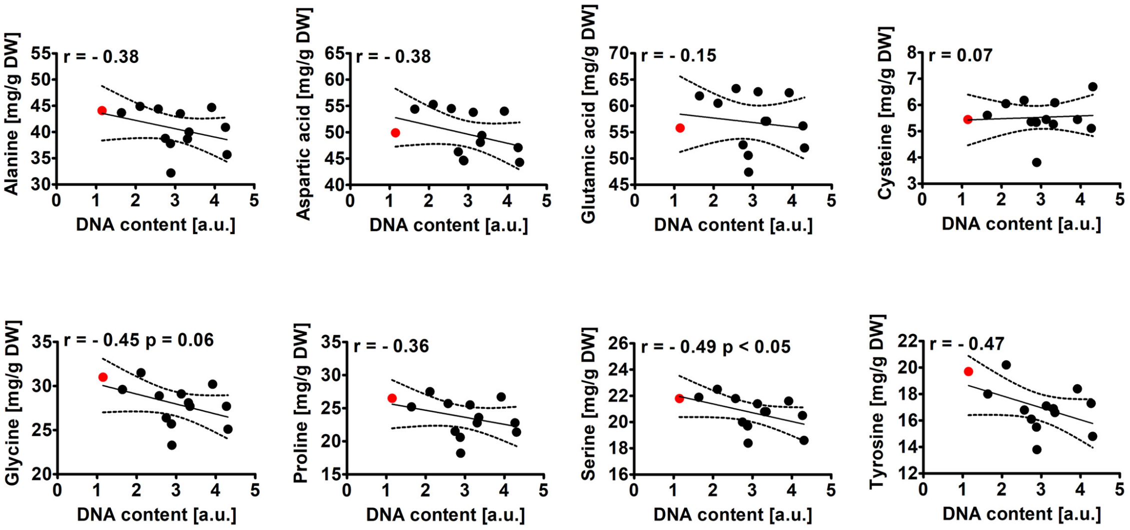

Supplementary Figure 3.

Colchicine and cytochalasin B-mediated effect on the levels of essential amino acids (top) and non-essential amino acids (bottom) in the microalga *Planktochlorella nurekis*. Correlation analysis between essential amino acids, non-essential amino acids and DNA content. Twelve CC-treated clones were considered (black dots). WT cells are denoted as a red dot. Results represent the mean from three independent experiments. The levels of amino acids [mg] were calculated per g of dry weight. The 95% confidence interval is shown. Correlation analysis of the data was performed using a Linear Correlation (Pearson r) test.
